# Structural and functional evidence for a substrate exclusion mechanism in mammalian tolloid like-1 (TLL-1) proteinase

**DOI:** 10.1016/j.febslet.2009.12.050

**Published:** 2010-02-19

**Authors:** Richard Berry, Thomas A. Jowitt, Laure Garrigue-Antar, Karl E. Kadler, Clair Baldock

**Affiliations:** a Wellcome Trust Centre for Cell-Matrix Research, Faculty of Life Sciences, University of Manchester, Michael Smith Building, Manchester M13 9PT, UK; b Laboratoire CRRET/UMR CNRS 7149, Facultė des Sciences, Universitė Paris-Est Crėteil Val de Marne (UPEC), 94010 CRETEIL Cedex, France

**Keywords:** Tolloid, Pro-collagen C-proteinase, Analytical ultracentrifugation, Chordin

## Abstract

Bone morphogenetic protein-1 (BMP-1)/tolloid proteinases are fundamental to regulating dorsal ventral patterning and extracellular matrix deposition. In mammals there are four proteinases, the splice variants BMP-1 and mammalian tolloid (mTLD), and tolloid like-1 and -2 (TLL-1/2). BMP-1 has the highest catalytic activity and lacks three non-catalytic domains. We demonstrate that TLL-1, which has intermediate activity, forms a calcium-ion dependent dimer with monomers stacked side-by-side. In contrast, truncated TLL-1 molecules having the same shorter structure as BMP-1 are monomers and have improved activity towards their substrate chordin. The increased activity exceeds not only that of full-length TLL-1 but also BMP-1.

**Structured summary:**

MINT-7386098: *BMP-1* (uniprotkb:P13497) *cleaves* (MI:0194) *Chordin* (uniprotkb:Q9H2X0) by *protease assay* (MI:0435)

## 1 Introduction

Bone morphogenetic protein-1 (BMP-1)/tolloid (TLD) family proteinases serve two main purposes; firstly the processing of extracellular matrix (ECM) precursors which range from structural components such as fibrillar collagens [1], enzymes [2], cellular anchoring proteins [3,4] and small leucine-rich proteoglycans [5,6]. Secondly, the release of transforming growth factor (TGF)-β superfamily members, including BMP-2 and -4, growth and differentiation factors (GDF) 8/11 and TGF-β1 from their latent complexes. Through these actions, BMP-1/TLD proteinases affect processes ranging from dorsal ventral patterning, growth of skeletal muscle, neurogenesis, to cellular behaviour [7–10].

**Fig. 1:**
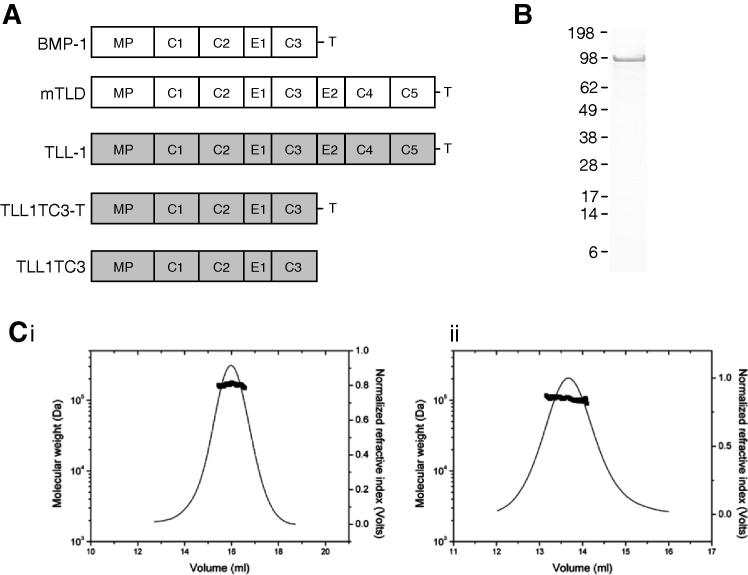
(A) Domain structures are displayed schematically. MP, metalloproteinase domain; C, CUB domain; E, EGF-like domain; T, C-terminal sequence. Protein derived from the *Bmpl* gene is shown in white and from the *Tll1* gene in grey. (B) Reducing SDS–PAGE of TLL-1, molecular weight markers in kDa are indicated. (C) MALLS analysis of TLL-1, in the presence of 1 mM CaCl_2_ the molecular mass was 219 500 Da (i) but in 2 mM EDTA it was 105 600 Da (ii). The theoretical molecular mass of TLL-1 (including purification tag) is 100 465 Da.

BMP-1/TLD proteinases have an N-terminal protease domain related to the digestive enzyme astacin [11] followed by non-catalytic CUB (Complement/Uegf/BMP-1) and epidermal growth factor-like (EGF) domains and a short C-terminal sequence (Fig. 1A). mTLD, TLL-1 and TLL-2 have 5 CUB and 2 EGF-like domains whereas BMP-1 lacks two CUB and one EGF C-terminal domains. There is evidence that the non-catalytic domains have a negative regulatory role [12,13] and we recently provided an explanation as to why mTLD is a poor proteinase relative to BMP-1 [14]. Our findings illustrated that BMP-1 is a monomer whereas mTLD forms a dimer in the presence of calcium-ions, which restricts its chordinase activity by a substrate exclusion mechanism. It is unknown whether this mechanism extends to other BMP-1/TLD family members, and in particular TLL-1 which is a more efficient proteinase than mTLD.

In the present report, we provide evidence that TLL-1 forms a dimer and show that truncated forms of TLL-1 having the same domain structure as BMP-1 are monomers, irrespective of the presence/absence of the unique C-terminal sequence. Surprisingly, the truncated TLL-1 molecules have higher chordinase activity not only than full-length TLL-1, but also BMP-1. Together these data provide evidence that the protease activity of TLL-1 is restricted by substrate exclusion.

## 2 Materials and methods

### 2.1 Expression and purification of recombinant proteins

TLL-1 was generated from an IMAGE clone comprising the first 1870 base pairs and the remaining 1169 base pairs synthesized by Genescript with a 3′ V5-His_(6)_ tag and ligated into the pCEP4 vector (Invitrogen) using Kpn1 and XhoI. TLL-1 molecules terminating after CUB3 with (TLL1TC3-T) and without (TLL1TC3) the C-terminal sequence, were amplified by PCR using TLL-1 and ligated into the pCEP-Pu vector using Not1 and Xho1. 293-EBNA cells were cultured and transfected as described in [14]. TLL-1 and variants were purified using a combination of nickel affinity and size exclusion chromatography as were BMP-1, mTLD and chordin as described in [14]. Enzyme and chordin concentrations were quantified based on comparison to known amounts of bovine serum albumin (BSA) using SDS–PAGE and GeneTools software (SynGene). A range of defined BSA quantities are visualised and a calibration curve plotted. The amount of enzyme in a known volume can then be determined.

### 2.2 Multi-angle laser light scattering (MALLS)

Samples (0.5 ml) were separated in 10 mM Tris–HCl (pH 7.4) containing 0.5 M NaCl in the presence of either 1 mM CaCl_2_ (Superdex-200 10/300 GL column) or 2 mM EDTA (Superdex-75 10/300 GL column) at 0.71 ml/min and passed through a Wyatt EOS 18-angle laser photometer with the 13th detector replaced with a Wyatt QELS detector. This was coupled to a Wyatt Optilab rEX refractive index detector and the hydrodynamic radius, molecular weight moments and concentration of peaks analysed using Astra5.3.2.

### 2.3 Analytical ultracentrifugation (AUC)

AUC experiments were performed using an XL-A ultracentrifuge as described in [14]. Equilibrium sedimentation was performed at 4 °C, using rotor speeds of 7, 12 and 19 000 rpm (35 670, 10 483 and 26 280 g) with scanning at 230 and 280 nm after equilibrium was reached (14 h). Association kinetics was performed using concentrations between 0.5 and 1.4 μM and data analysed using Sedphat [15]. Velocity sedimentation was performed at 40 000 rpm (116 480 g) at 20 °C (TLL-1) or 45 000 rpm (147 420 g) at 10 °C (TLL1TC3 variants) with the sedimenting boundary monitored every 90 seconds (TLL-1) or 3 min (TLL1TC3 variants) for 200 scans. Protein concentrations were 174 μg/ml (TLL-1), 59 μg/ml (TLL1TC3), 47 μg/ml (TLL1TC3-T in Ca^2+^) and 196 μg/ml (TLL1TC3-T in EDTA). Data was interpreted using Sedfit [16]. Bead models were generated using the atomic coordinates of homologous domains in the modeling software SOMO [17].

### 2.4 TEM

TLL-1 (10 μg/ml) in the presence of 1 mM CaCl_2_ was absorbed onto EM grids and stained with 4% (w/v) uranyl acetate and observed in an FEI Tecnai12 TEM at 120 keV. Images were recorded on a CCD camera at 69 000× magnification between 0.5 and 1.5 μm defocus and processed using Imagic5 software [18]. Four hundred and eighty-five particles were band-pass filtered with high and low frequency cut-offs of 20 Å and 180 Å.

### 2.5 Activity assays

Purified chordin (2 μg) was incubated in the presence or absence of 30 ng enzyme in 50 mM Tris–HCl (pH 7.4) containing 150 mM NaCl and 5 mM CaCl_2_ at 37 °C for 4 h [19]. Reactions were stopped by adding LDS sample buffer (Invitrogen) and heating to 95 °C for 5 min. Reaction products were separated by SDS–PAGE and visualised by silver staining. Chordinase assay products were quantified by densiometry using SynGene software.

## 3 Results

Purified TLL-1 (Fig. 1B) eluted from SEC-MALLS in buffer containing calcium as a single peak with a mass of 219 500 Da (Fig. 1C(i)) suggesting TLL-1 is a dimer in solution. To determine whether the TLL-1 dimer requires the presence of calcium, we analysed samples in the presence of EDTA (Fig. 1C(ii)). Under these conditions, the mass was reduced to 105 600 Da, in good agreement with a monomer (Table 1). The strength of TLL-1 self-association was assessed using sedimentation equilibrium AUC (data not shown). TLL-1 was predominantly dimeric with very strong self-association (*K*_d_ = 2 nM at 0.5 mM [CaCl_2_]).

**Fig. 2:**
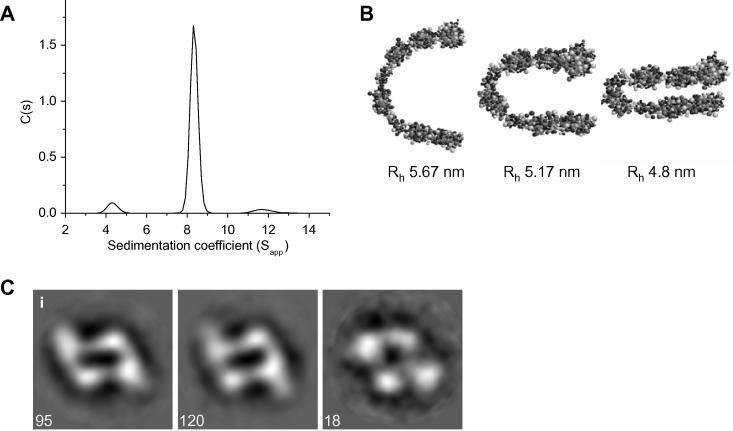
(A) Sedimentation velocity AUC of TLL-1 in the presence of 1 mM CaCl_2_. The majority of protein is present as a dimer at 8.3 *S*_app_. (B) Bead models generated for the TLL-1 monomer. A horseshoe-like conformation gives the closest fit to experimental *R*_h_ of 5.33 nm. (C) The TLL-1 dimer was investigated using single particle TEM. (i) Three representative classes are shown; the number of particles in each class is indicated (box size = 21.6 nm). Potential dimer packing models are shown in more detail in [14].

To investigate the dimeric arrangement of TLL-1, we used sedimentation velocity AUC. In the presence of 1 mM calcium-ions, the predominant species has an *S*_20,W_ of 8.88 (Fig. 2A). This species has a mass estimate of 196 000 Da, similar to that expected for a dimer. To gain insight into the structure of the TLL-1 monomer we generated bead models and a horseshoe-like conformation (Fig. 2B) gave the best fit to the experimentally-derived hydrodynamic radius (*R*_h_) of 5.33 nm (Table 1). We used single particle TEM to investigate the arrangement of the dimer, following classification it was evident that TLL-1 particles were present in a single orientation showing side-by-side stacking of monomers (Fig. 2C).

**Fig. 3:**
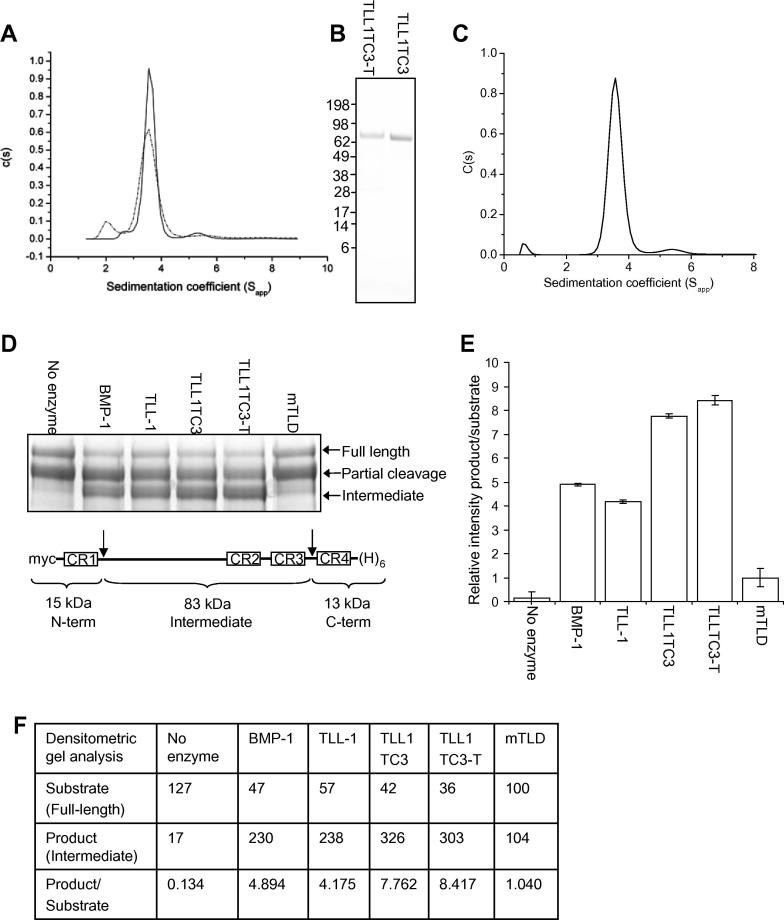
(A) Sedimentation velocity AUC of TLL1TC3-T in the presence of 1 mM CaCl_2_ (solid line) and 2 mM EDTA (dotted line). The peak at 3.5 *S*_app_ corresponds to the monomer. (B) Non-reducing SDS–PAGE of truncated TLL-1 variants. Molecular weight markers in kDa are indicated. (C) TLL1TC3 appears almost identical to TLL1TC3-T when analysed by sedimentation velocity AUC. (D) Silver stained SDS–PAGE of chordin following incubation with enzymes, the chordin domain structure is shown (CR, cysteine-rich repeat; myc, c-myc epitope; (H)_6_, poly-histidine tag; arrows indicate cleavage sites). (E) Densitometric analysis of chordin cleavage is shown for each enzyme represented as the amount of product (intermediate fragment) relative to substrate (full-length chordin). Data are the mean of three independent experiments ± S.E. (F) Table showing the values from the densitometric analysis used to plot (E).

To investigate if TLL-1 is restricted by a substrate exclusion mechanism we made a TLL-1 variant with the same domain structure as BMP-1 (TLL1TC3-T with unique C-terminal sequence). Due to aggregation we were unable to obtain a mass for TLL1TC3-T using MALLS, therefore we analysed protein samples by sedimentation velocity AUC. In the presence of EDTA or calcium, the c(s) profile are the same with a predominant species at 3.5 *S*_app_ (Fig. 3A) suggesting almost all protein is monomeric (Table 1). Since there are no species at higher (>4) *S*_app_ in samples containing calcium, we conclude that TLL1TC3-T does not form calcium-ion dependent dimers.

In a previous report, a construct analogous to TLL1TC3-T lacking the C-terminal sequence formed oligomeric aggregates observed on non-reducing SDS–PAGE thought to be due to misfolding [12]. To test this possibility, we generated TLL1TC3 which lacks the C-terminal sequence. Neither variant (with or without the C-terminal sequence) showed evidence of aggregation (Fig. 3B). To confirm the oligomeric status of TLL1TC3 we used sedimentation velocity AUC. In the presence of calcium-ions the c(s) profile is almost identical to that of TLL1TC3-T (Fig. 3C), suggesting that TLL1TC3 is also monomeric.

To determine the proteolytic activity of the TLL-1 variants, we compared their chordinase activity to that of mTLD, BMP-1 and TLL-1 (Fig. 3D and E). Chordinase activity was detected for mTLD but this was considerably lower than that of BMP-1 and TLL-1, which appear to have similar levels of chordinase activity. However, the truncated forms of TLL-1 are approximately twice as efficient as TLL-1 at processing chordin.

## 4 Discussion

In this study we find that TLL-1 forms a calcium-dependent dimer via a side-by-side stacking of monomers and that the dimer has a much tighter association than mTLD. We also show that the region responsible for self-association resides in the C-terminal three domains. These findings suggest dimerisation is most likely a feature of all mammalian BMP-1/TLD family members that have EGF2, CUB4 and CUB5. Based on these data, we believe that the proteolytic activity of the TLL-1 dimer is restricted by a substrate exclusion mechanism that requires the presence of the C-terminal domains. This conclusion is based on the observations that monomeric forms of TLL-1 lacking the C-terminal domains are more efficient chordinases than the full-length molecule and these “BMP-1 forms” of TLL-1 are also more efficient chordinases than BMP-1. This latter observation suggests that differences in activity between TLL-1 and mTLD are due to variations in amino acid residues rather than gross mechanistic differences.

This is further supported by an independent study in which the proteinase activity of a construct analogous to TLL1TC3-T was investigated [12]. This molecule was described as being more efficient than full-length TLL-1 at cleaving procollagen I and probiglycan. However, what was also apparent in their results, is that this molecule was also more efficient than BMP-1. These data support our findings and suggest the substrate exclusion mechanism that restricts the activity of TLL-1 extends to other substrates.

We also find that the presence or absence of the C-terminal sequence has little effect on the chordinase activity of truncated TLL-1 molecules. In a previous report, it was observed that a molecule analogous to TLL1TC3 was a poor C-proteinase whereas with the C-terminal sequence it was highly efficient [12]. Since the form lacking the C-terminal sequence was observed to form aggregates, these differences were thought to be due to misfolding, and it was hypothesised that the C-terminal sequence was required for correct folding. In contrast, our studies reveal the presence or absence of the C-terminal sequence has no effect on the oligomeric status or chordinase activity of these molecules. These discrepancies can be explained by small differences in the CUB3 domain. In the previous report, the molecule terminated after *K*^724^. Our domain boundary was determined by analysis of the intron-exon boundary and contained an additional six amino acids (up to *D*^730^). Presumably these aided in the correct folding of our molecules.

The difference in activity between mTLD and TLL-1 could be due to subtle differences in the intrinsic affinity of the BMP-1/mTLD and TLL-1 protease domains for substrates. In support of this, chordin cleavage by the isolated protease domains from BMP-1/mTLD and TLL-1 showed that the TLL-1 domain is significantly more efficient [12]. Alternatively, differences in the affinity of the non-catalytic domains for chordin may provide an explanation. Despite the similarities in overall architecture, the strength of TLL-1 self-association is greater than mTLD. It is conceivable that this may result in different patterns of activity in vivo. The expression of *Bmpl* and *Tll1* overlap in a number of tissues [20], differences in self-association could favour the effect of one proteinase depending on the calcium and/or protein concentrations present. Our findings also raise the possibility of mTLD/TLL-1 heterodimers, which could add another layer of complexity.

## Figures and Tables

**Table 1:** Data was collected by (*a*) MALLS, (*b*) AUC: molecular weight (MW) in Daltons; hydrodynamic radius (*R*_h_) in nm; sedimentation coefficient (*S*_20,w_) and frictional coefficient (*f*/*f*_0_). Theoretical MWs (including purification tag) are 100 465 Da (TLL-1), 68 341 Da (TLL1TC3-T) and 67 113 Da (TLL1TC3). ND = not determined.

	MW_*a*_	MW_*b*_	*R*_h,*b*_	*S*_20,W_	*f*/*f*_o_
TLL-1 Ca^2+^	219 500 ± 659	196 000	5.52	8.88 ± 0.15	1.44
		73 900	5.33	4.59 ± 0.21	1.75
TLL-1 EDTA	105 600 ± 12 672	ND	ND	ND	ND
TLL1TC3 Ca^2+^	ND	67 100	3.36	4.91 ± 0.33	1.26
TLL1TC3-T Ca^2+^	ND	65 990	3.55	4.73 ± 0.38	1.32
TLL1TC3-T EDTA	ND	67 200	3.67	4.59 ± 0.32	1.36
